# Supplementary material for: End Sequence Analysis Toolkit (ESAT) expands the extractable information from single-cell RNA-seq data
Source: Genome Res. 2016 Oct;26(10):1397–410. doi: 10.1101/gr.207902.116 (PMC5052061; doi:10.1101/gr.207902.116)
Supplement: Supplemental Material [file supp_gr.207902.116_Supp_Materials.tar.gz › ESAT-esat_v0.1/doc/html/classbroad_1_1core_1_1sequence_1_1_sequence_utils.html]

ESAT: broad.core.sequence.SequenceUtils Class Reference


ESAT


- Main Page
- Related Pages
- Packages
- Classes
- Files

- Class List
- Class Index
- Class Hierarchy
- Class Members

All Classes Namespaces Files Functions Variables Enumerator Pages

- broad
- core
- sequence
- SequenceUtils

Static Public Member Functions |
List of all members

broad.core.sequence.SequenceUtils Class Reference

|  |  |
| --- | --- |
| Static Public Member Functions | |
| static Sequence | getChrSequence (String seqDir, String chr) throws IOException |
|  | |
| static String | getChrSizesFile (String seqDir) |
|  | |
| static ContinuousDataAlignmentModel | getDataModel (String bamFile, String sizeFile, String chr, boolean removeDuplicates) throws IOException |
|  | |
| static ContinuousDataAlignmentModel | getDataModel (String bamFile, String sizeFile, boolean removeDuplicates) throws IOException |
|  | |
| static ContinuousDataAlignmentModel | getDataModel (String bamFile, String sizeFile, int minMappingQuality, boolean removeDuplicateFlags, boolean weighReadCounts, String strand, boolean loadPairsAsFragments) throws IOException |
|  | |

## Member Function Documentation

|  |  |  |  |
| --- | --- | --- | --- |
| static Sequence broad.core.sequence.SequenceUtils.getChrSequence | ( | String | *seqDir*, |
|  |  | String | *chr* |
|  | ) |  | throws IOException |

static

|  |  |  |  |  |  |
| --- | --- | --- | --- | --- | --- |
| static String broad.core.sequence.SequenceUtils.getChrSizesFile | ( | String | *seqDir* | ) |  |

static

|  |  |  |  |
| --- | --- | --- | --- |
| static ContinuousDataAlignmentModel broad.core.sequence.SequenceUtils.getDataModel | ( | String | *bamFile*, |
|  |  | String | *sizeFile*, |
|  |  | String | *chr*, |
|  |  | boolean | *removeDuplicates* |
|  | ) |  | throws IOException |

static

Here is the caller graph for this function:

|  |  |  |  |
| --- | --- | --- | --- |
| static ContinuousDataAlignmentModel broad.core.sequence.SequenceUtils.getDataModel | ( | String | *bamFile*, |
|  |  | String | *sizeFile*, |
|  |  | boolean | *removeDuplicates* |
|  | ) |  | throws IOException |

static

Here is the call graph for this function:

|  |  |  |  |
| --- | --- | --- | --- |
| static ContinuousDataAlignmentModel broad.core.sequence.SequenceUtils.getDataModel | ( | String | *bamFile*, |
|  |  | String | *sizeFile*, |
|  |  | int | *minMappingQuality*, |
|  |  | boolean | *removeDuplicateFlags*, |
|  |  | boolean | *weighReadCounts*, |
|  |  | String | *strand*, |
|  |  | boolean | *loadPairsAsFragments* |
|  | ) |  | throws IOException |

static

---

The documentation for this class was generated from the following file:

- java/broad/core/sequence/SequenceUtils.java


---

Generated on Fri Aug 15 2014 15:10:56 for ESAT by  

 1.8.7
